# Measurement of mobility and lifetime of electrons and holes in a Schottky CdTe diode

**DOI:** 10.1088/1748-0221/9/12/C12032

**Published:** 2014-12-01

**Authors:** G. Ariño-Estrada, M. Chmeissani, G. de Lorenzo, M. Kolstein, C. Puigdengoles, J. García, E. Cabruja

**Affiliations:** a Institut de Física d’Altes Energies, IFAE, 08193 Bellaterra, Barcelona, Spain; b Centro Nacional de Microelectrónica, IMB-CNM (CSIC), 08193 Bellaterra, Barcelona, Spain

**Keywords:** Solid state detectors, Charge transport and multiplication in solid media, Detector modelling and simulations II (electric fields, charge transport, multiplication and induction, pulse formation, electron emission, etc)

## Abstract

We report on the measurement of drift properties of electrons and holes in a CdTe diode grown by the travelling heating method (THM). Mobility and lifetime of both charge carriers has been measured independently at room temperature and fixed bias voltage using charge integration readout electronics. Both electrode sides of the detector have been exposed to a ^241^Am source in order to obtain events with full contributions of either electrons or holes. The drift time has been measured to obtain the mobility for each charge carrier. The Hecht equation has been employed to evaluate the lifetime. The measured values for *μτ*_*e/h*_ (mobility-lifetime product) are in agreement with earlier published data.

## 1 Introduction

CdTe and CdZnTe detectors are nowadays a good choice for gamma ray detection due to their excellent energy resolution, easy segmentation into voxels and relatively good detection efficiency. When the ionizing radiation interacts with the bulk, a cloud of free charged particles is created, either with negative charge, the electrons, or with positive charge, the holes. Due to the bias voltage applied on the semiconductor electrodes, the charge carriers drift in opposite directions. Their velocity depends on the magnitude of the bias voltage and the structure of the crystal lattice of the material. The mobility (*μ*) is the velocity normalized to the bias voltage applied on the semiconductor. The large difference between the hole and the electron mobilities (*μ*_*h*_ and *μ*_*e*_, respectively) for CdTe and CdZnTe increases the time walk and hence the timing resolution. This timing limitation is one of the main drawbacks of using CdZnTe in fast triggering schemes such as Time Of Flight (TOF).

The charge carriers can be trapped in the lattice due to impurities or recombinations, resulting in a total charge loss. The lifetime (*τ*) is the average time for a charge carrier to get trapped in the material. The higher the level of impurities in the material, the shorter *τ* becomes and more trapping effect is observed. The product of *μ* and *τ* is called the trapping length, *l* = *μτ*, and is an estimation of how long the charge carrier can move among the material without being trapped.

The amount of charge loss can be evaluated if the values of *μ* and *τ* for both charge carriers and the depth of interaction point are known using the Hecht relation [1, 2]. The charge induced on the electrodes can be obtained with the Schockley-Ramo theorem [3, 4], in particular, with the solution for a rectangular detector. The drift can be discretized and the charge loss and the induced charge can be updated for each point to obtain a charge vs time curve on the electrodes for a non perfectly coplanar detector.

The knowledge of these parameters allows one to predict with precision the shape of the charge induced on the electrodes and optimize the signal processing electronics coupled to the semiconductor. Additionally, they can be used to reconstruct the charge loss on each event or the delay on the trigger time and improve the energy and time detection resolution. In the VIP Project [5, 6] we are interested in the fine tuning of the signal processing electronics and the correction to improve the energy and time resolution. For this purpose, we have measured *μ* and *τ* for both charge carriers. The drift time has been measured to evaluate the *μ*, whereas, a fit of the outcoming signal has been used to evaluate the *τ*. The work concludes with a comparison of previously presented results.

## 2 Experimental method

**Figure 1:**
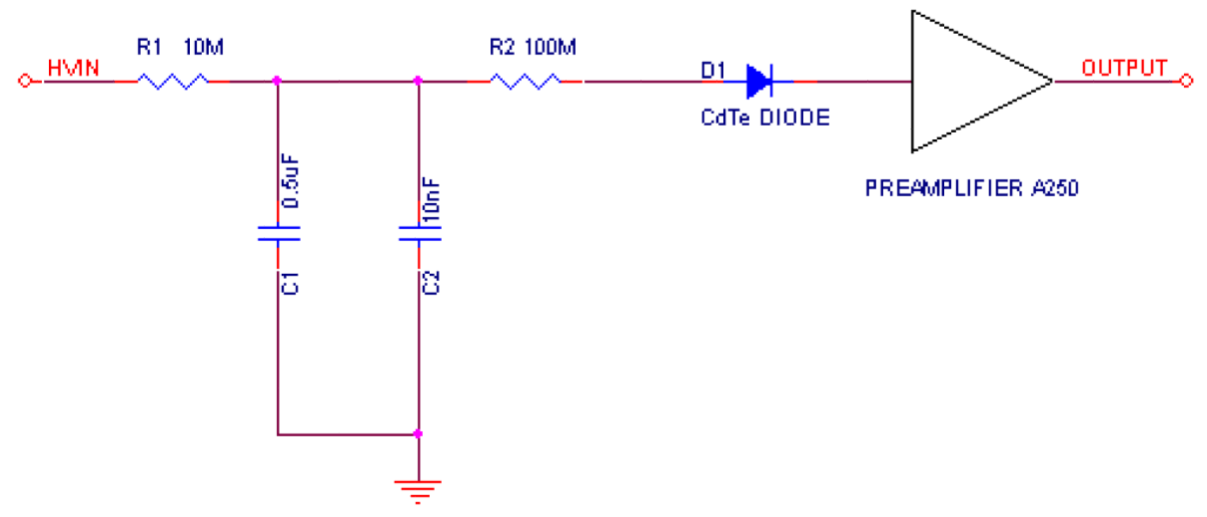
Schematic of the circuit with the RC filter, the CdTe diode and the preamplifier.

A CdTe diode manufactured by ACRORAD has been used. It is a Schottky diode with the cathode of Pt, the anode of Au/Ti/Al and with dimensions 10 mm × 10 mm × 2 mm. It has been placed in a vertical position, on one of its lateral sides, in order to be bombarded with alpha particles, on either side of the anode or the cathode. The radiactive source used has been ^241^Am, which emits alpha particles with an energy of 5.5 MeV. A two stage filter RC has been placed between the high voltage power supply and the diode as depicted in figure 1.

Charge integration has been used for the detector readout. A charge sensitive amplifier (preamplifier) A250 from AMPTEK has been coupled to the detector output. The preamplifier output has been recorded with an oscilloscope TDS3024B from Tektronix. A Labview executable has been used to acquire the full charge vs time curve for each event. The diode has been biased with a Keithley 2410 power supply. In order to avoid the polarization effect of the bias voltage in the diode, it has been ramped down every 100 s and kept at 0 V for 30 s and then ramped up again to −500 V.

## 3 Data analysis

### 3.1 Mobility

The mobility of a charge carrier can be evaluated when the detector thickness (D), the bias voltage applied (V) and its drift time along the diode (Δ t) are fixed using equation (3.1). Given that D and V are known, a measurement of Δ t is enough to obtain a value for *μ*.

(3.1) $$\mu =\frac{D^{2}}{V\Delta t}$$

**Figure 2:**
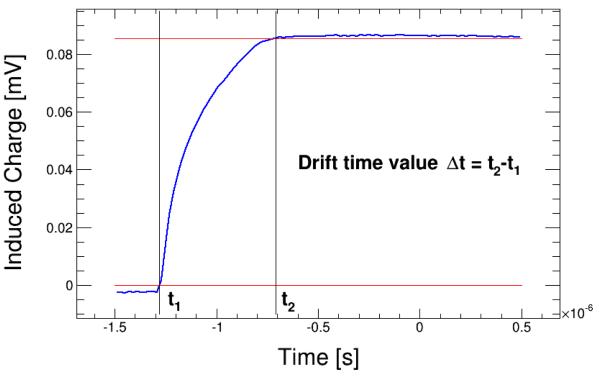
Example of the drift time measurement on one event.

An example of the drift time evaluation of event is shown in figure 2.

### 3.2 Lifetime

The charge induction curve for each event has been fit to obtain an average value of *τ*. The function used for the fit is a combination of the Hecht equation (3.2), in order to include the charge trapping effect, and the solution for the weighting potential for a rectangular geometry using the method of images [2] to predict the charge induced in the electrodes by the charge carriers as a function of time.

(3.2) $$\frac{Q*}{Q}=\frac{\nu_{h}\tau_{h}^{*}}{D}\left(1-exp\left(\frac{-x_{i}}{\nu_{h}\tau_{h}^{*}}\right)\right)+\frac{\nu_{e}\tau_{e}^{*}}{D}\left(1-exp\left(\frac{x_{i}-D}{\nu_{e}\tau_{e}^{*}}\right)\right)$$

**Figure 3:**
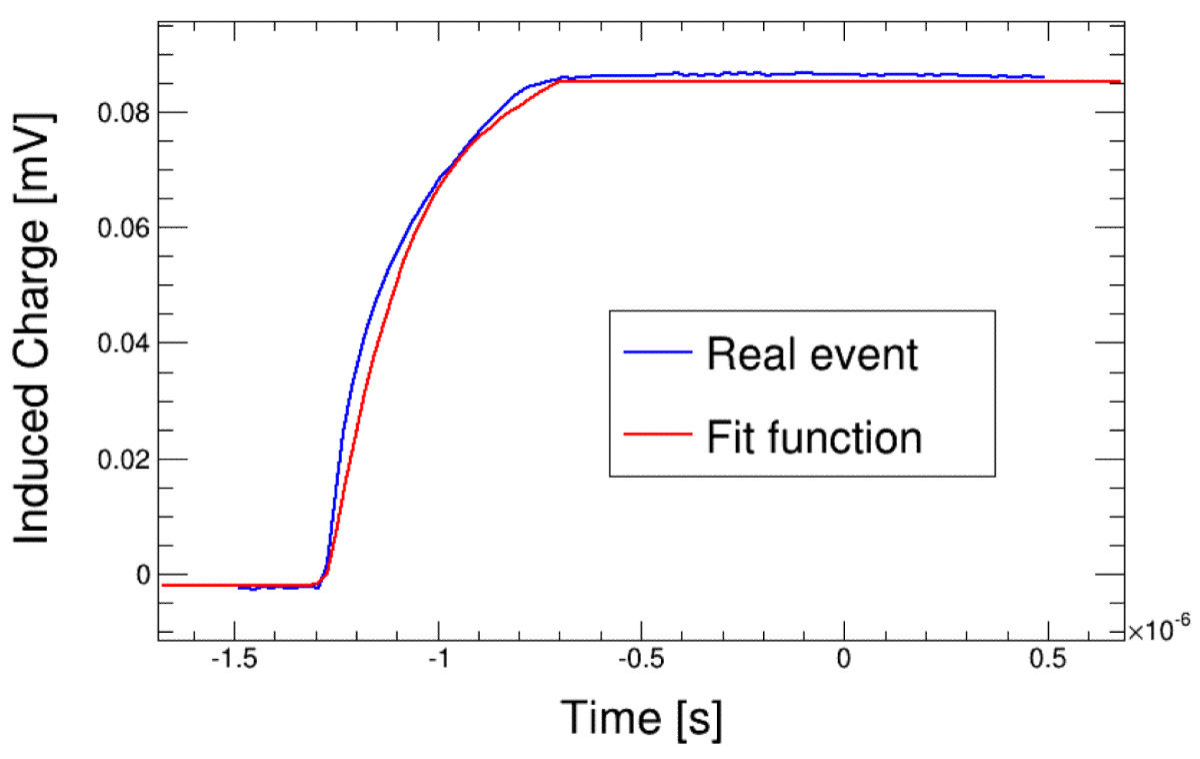
Example of the fit function on the curve of one event.

The result is a function of the time (*t*), the depth of interaction point (*x*_0_) and *μ* and *τ* for each charge carrier, *f*(*t*, *x*_0_,*μ_e_*,*τ_e_*,*μ_h_*,*τ_h_*). For events with full contribution of electrons or holes *x*_0_ is 0 or D respectively and the dependency of the function becomes *f*(*t*, *μ*_*e\h*_,*τ*_*e\h*_). The value of *μ*_*e\h*_ has been previously determined with the aforementioned method so it becomes a function with one variable, *t*, and one parameter, *τ*_*e\h*_. The example of an event with the result fit function is shown in figure 3. The result of the fits for all the events will give a distribution of this parameter for each charge carrier.

## 4 Results

**Figure 4:**
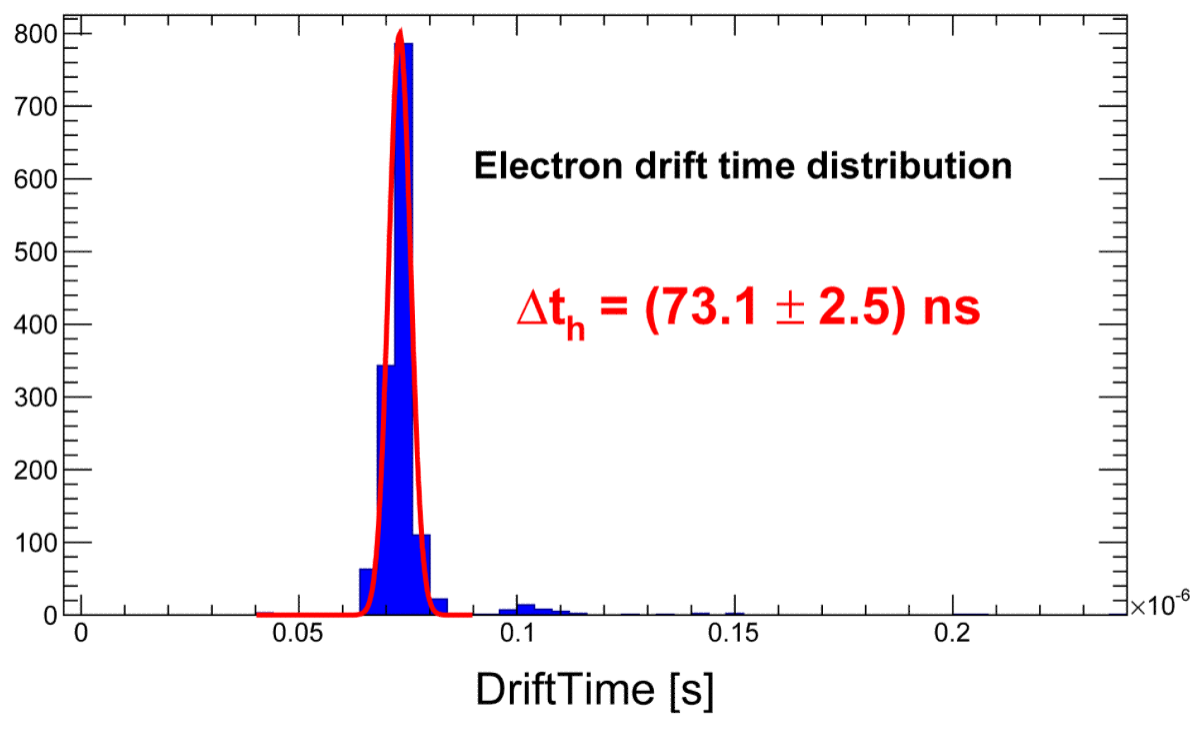
Drift time distribution for electrons in a CdTe diode of 2 mm thickness operated at −500 V.

**Figure 5:**
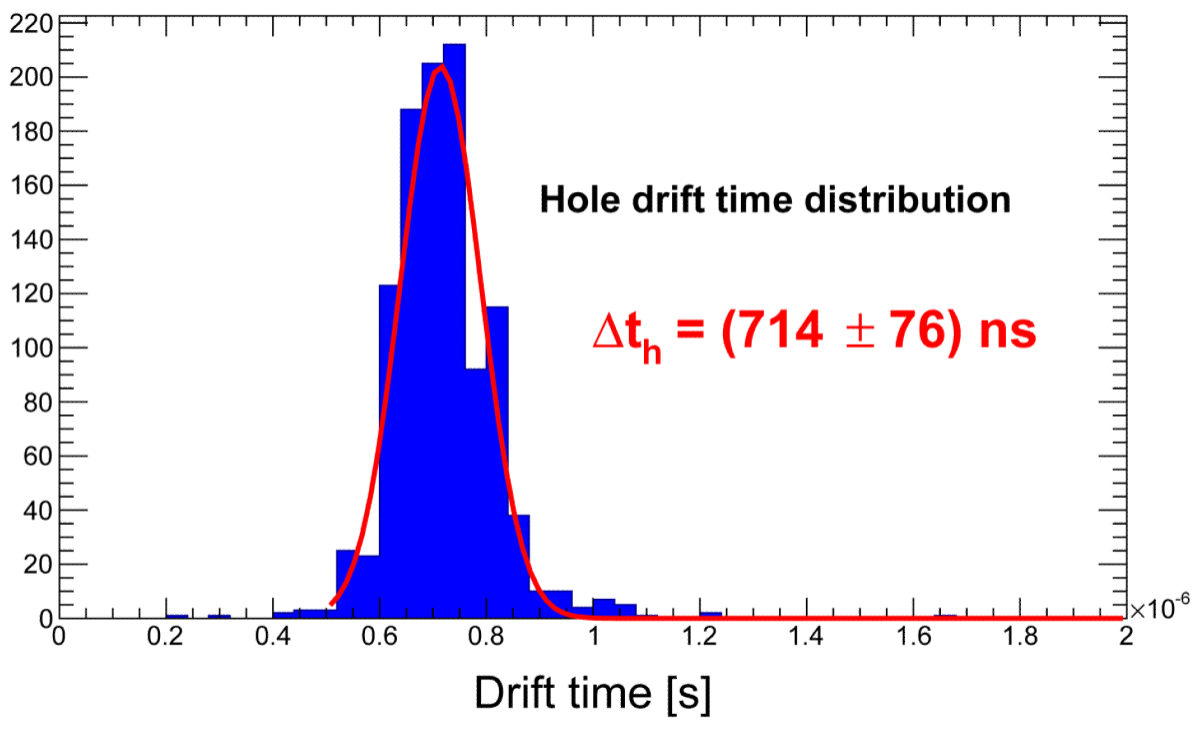
Drift time distribution for holes in a CdTe diode of 2 mm thickness operated at −500 V.

The distribution of the drift time for electrons and holes are shown in figures 4 and 5 respectively. The expression (3.1) has been used to obtain the mobilities with the average drift times measured and D=0.002 m and V=500 V. The values obtained are *μ*_*e*_=(1090±40) cm^2^/(V·s) and *μ*_*h*_=(110±10) cm^2^/(V·s).

**Figure 6:**
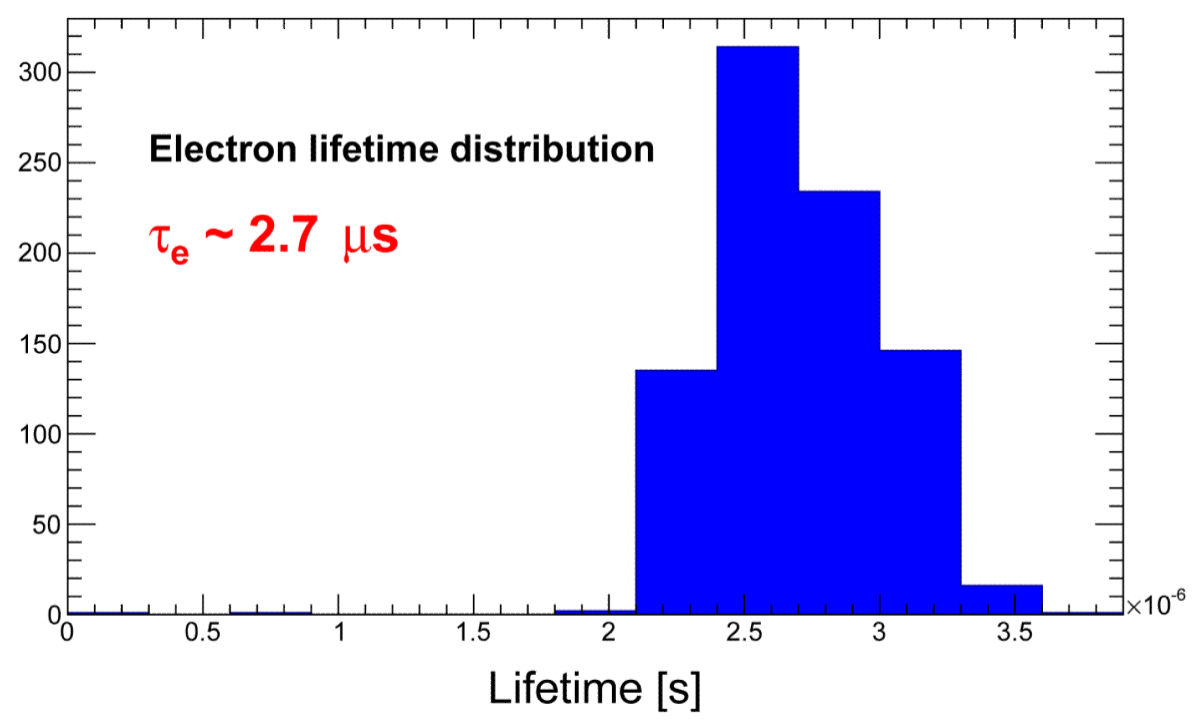
Lifetime distribution for electrons.

**Figure 7:**
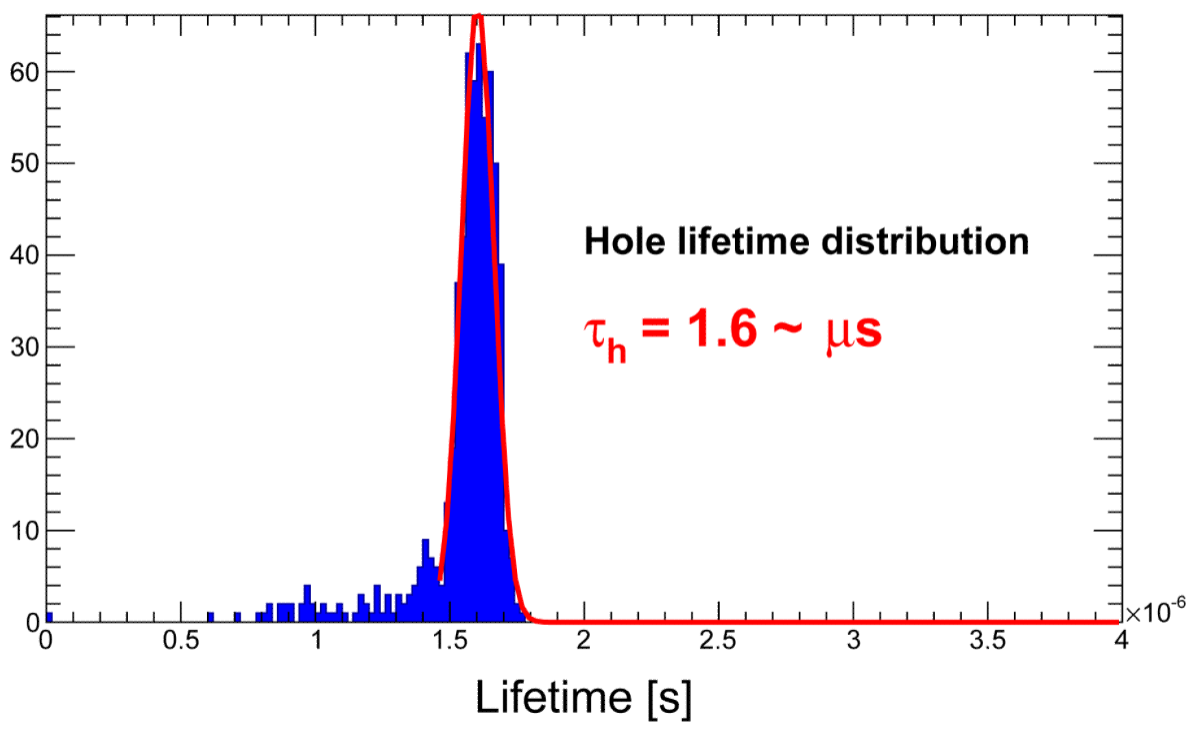
Lifetime distribution for holes.

The distributions of *τ* for electron and holes are shown in figures 6 and 7 respectively. One can see that the distribution for electrons is wider than that of the holes. This is created by the fact that the electrons are less affected by the charge trapping and the outcoming value from the fit is less precise. With these values one can easily compute the approximate values of the mobility-lifetime products *μτ*_*e*_ ~ 2.7·10^−3^m^2^/(V·s) and *μτ*_*h*_ ~ 1.8·10^−4^m^2^/(V·s).

In table 1 the measurements of *μ* and *μτ* are summarized next to some previously published results.

## 5 Conclusion

A setup to measure the mobility and lifetime of electrons and holes has been built. It consists of a CdTe diode coupled to a preamplifier. Both electrodes have been exposed to a ^241^Am source to detect alpha particles and the outcoming signals have been recorded with an oscilloscope. The measured drift time and mobility values of *μ*_*e*_ =(1090±40) cm^2^/(V·s) and *μ*_*h*_=(110±10) cm^2^/(V·s) have been obtained.

The same curves have been fit to obtain the *τ* values. For holes, *τ*_*h*_ ~ 1.6 whereas for electrons *τ*_*e*_~2.7. The *μτ* products are approximately *μτ_e_*~2.9·10^−3^m^2^/(V·s) and *μτ*_*h*_~1.8·10^−4^m^2^/(V·s) which are similar to those measured for CdTe diodes in [9, 10].

The values obtained in this work are those expected for CdTe as one can compare with other publication with the values shown in table 1. Further analysis will be carried out in the VIP project based on this work

## Figures and Tables

**Table 1:** Values of *μ* and *μ*τ for electrons and holes shown in previous complications and those obtained in this work

Reference	*μ_e_*[cm^2^/(V·s)]	*μ_h_* [cm^2^/(V·s)]	*μ_e_*τ*_e_* [10^−3^ cm^2^/V]	*μ_h_*τ*_h_* [10^−4^ cm^2^/V]

[7]	1000-1100	100	-	-
[8]	1040	70	-	-
[9]	-	-	2	0.8
[10]	-	-	2.8	0.9
[11]	946±50	79.5±9	1-2	1
[12]	1100	100	3	2
[13]	1050	80	~1	~0.5-1
Our Approach	1090	110	2.7	1.8
